# Non-utilisation of health care services during the COVID-19 pandemic: Results of the CoMoLo study

**DOI:** 10.25646/9564

**Published:** 2022-03-16

**Authors:** Christin Heidemann, Lukas Reitzle, Christian Schmidt, Judith Fuchs, Franziska Prütz, Christa Scheidt-Nave

**Affiliations:** Robert Koch Institute, Berlin, Department of Epidemiology and Health Monitoring

**Keywords:** UTILISATION, CARE, PHYSICIAN VISIT, TREATMENT, COMPLAINTS, TELEMEDICINE, COVID-19, SARS-COV-2

## Abstract

Based on data from the CORONA-MONITORING lokal (CoMoLo) study conducted in four municipalities particularly affected by the COVID-19 pandemic, this article investigates the non-utilisation of health care services in the population aged 18 years and older (n=9,002) in relation to the period after the introduction of the containment measures in March 2020. The results show that about one third of the respondents (35.5%) gave up at least one of the surveyed health care services. The most frequent cancellations were dental (15.2%) and specialist check-ups (11.8%), followed by postponement of physiotherapy, ergotherapy or speech therapy (6.1%), cancellation of general practitioner (GP) check-ups (5.8%), postponement of psychotherapy (2.0%), postponement of planned hospital treatment (1.8%) and not going to an emergency room (0.7%). Almost 10% of the respondents reported not visiting a physician despite health complaints. Compared to respondents without such a waiver, these respondents were more often female and younger than 35 years, less often rated their health as very good or good, more often had a diagnosis of depression and more often used telemedical contacts as an alternative to visiting the practice during the pandemic. Further analyses of trends in utilisation behaviour and changes in health status over the course of the COVID-19 pandemic are important.

## 1. Introduction

Since the emergence of the first series of SARS-CoV-2 cases in China at the end of 2019 [1], the novel corona virus, that can lead to COVID-19 disease with sometimes severe courses [2], has been spreading worldwide. In the last week of January 2020, there was the first laboratory-confirmed COVID-19 case in Germany. The high infection dynamics led to every federal state reporting COVID-19 cases as early as the second week of March 2020 [3, 4]. As a result, comprehensive non-pharmaceutical measures to contain SARS-CoV-2 infection were introduced at the federal and state levels before the end of March 2020. These included population-based measures such as general contact restrictions (e.g. by banning events, closing educational institutions and allowing telephone sick notes) and individual infection hygiene measures such as maintaining a minimum distance and wearing mouth-to-nose coverings [3, 4]. Other measures included strengthening intensive care and ventilation capacity in hospitals for COVID-19 patients by postponing scheduled procedures, which was necessary in some cases [5]. The extent and time limit of these measures depended on the development of the pandemic situation. Thus, from the end of April 2020, the measures were gradually relaxed, and in November 2020 they were strengthened again [4].

So far, initial evaluations of the effects of the COVID-19 pandemic and the introduced containment measures on the health care situation of the population are available for the year 2020 [6, 7]. For example, accounting data from the Associations of Statutory Health Insurance (SHI) Physicians show that the number of medical and psychotherapeutic outpatient services in 2020 fell significantly across almost all specialities from March onwards compared to the corresponding period of the previous year, only returning to normal in May and in some cases falling again in the third and fourth quarters [8]. For inpatient services, based on AOK acounting data, a decline is also described for 2020 in comparison to the previous year, which was more pronounced in terms of numbers in the period from March to May than in the period from October to December and mainly, but not exclusively, applied to postponable treatment events [9, 10]. A decline in the number of emergency department visits was also observed with the start of containment measures in 2020 based on a multi-centre data collection in emergency departments [11]. The reasons for the decline in treatment cases are not exclusively attributed to shifts or changes in the supply of care due to COVID-19 cases requiring priority treatment, but at least partly also to fears of infection with SARS-CoV-2 [6].

The analyses from the point of view of the service providers only allow limited conclusions to be drawn about the need in the population. For example, results from the nationwide, population-based survey German Health Update (GEDA) of the Robert Koch Institute (RKI) also indicate a decline in self-reported utilisation of general and specialist outpatient services during the first phase of the containment measures compared to the previous year [12]. However, an evaluation of the GEDA data focussed on persons with diabetes shows at least no relevant change in general practitioner (GP) utilisation for this group of respondents with regularly required GP visits [13]. Similarly, results from the COVID-19 Snapshot Monitoring (COSMO) online survey indicate that a substantial proportion of participants postponed potentially deferrable preventive care appointments due to the COVID-19 pandemic, but the majority considered a supply of necessary physician visits and medications to be assured during the phases of containment activities in 2020 [14–16].

This analysis uses population-based data from the CORONA-MONITORING lokal (CoMoLo) study conducted by the RKI in the second to fourth quarter of 2020 to explore the following questions: 1) What proportion of respondents was affected by suspended health services in the different health care settings with regard to the period of strict containment measures since March 2020, and 2) How do people who refrained from health care services despite health complaints differ from those who did not?

## 2. Methodology

### 2.1 CORONA-MONITORING lokal (CoMoLo) study

CoMoLo is a population-based seroepidemiological study that was conducted in four municipalities in Germany that were particularly affected by the COVID-19 pandemic as cross-sectional surveys in independent samples [17]. Criteria for selecting the study sites were a cumulative SARS-CoV-2 reporting incidence of more than 500 cases per 100,000 persons of the population before the start of the study and a willingness to cooperate on site. The aim was to determine the seroprevalence (i.e. the proportion of the population with previous SARS-CoV-2 infection overall), to differentiate the proportion of undetected or asymptomatic SARS-CoV-2 infections, and to determine the risk factors for infection. A further concern was to record changes in health behaviour, health status and the use of health care services in the context of the pandemic and the containment measures introduced [18].

The cross-sectional surveys were based on randomly drawn population registration office samples in Kupferzell (Baden-Württemberg), Bad Feilnbach (Bavaria), Straubing (Bavaria) and Berlin-Mitte [17]. Adults aged 18 years and older who were registered with their main residence in the respective municipality during the survey period were included. An oropharyngeal swab, a blood sample and an interview based on a short written questionnaire were performed at a temporary study centre with examination buses or during a home visit. Participants were asked for permission to re-contact for a detailed follow-up interview. Approximately one to two weeks after participation in the study, an in-depth follow-up interview was conducted, either webbased or by telephone [18, 19].

More information on reporting incidence, sampling and study methodology as well as results on seroprevalence and magnitude of undetected or asymptomatic cases per study site are given elsewhere [20–25]. Table 1 summarises the timing, local infrastructure and local outbreak events as well as participant numbers, response rates and sociodemographic characteristics for each study site.

### 2.2 Data collection

#### Health care services

The non-utilisation of health care services, which is the focus of this article, was assessed through the web-based or telephone CoMoLo follow-up interview via the following question: ‘18 March 2020 is a key date that we refer to frequently in this questionnaire. On that day was the Chancellor’s speech in which she recommended the nationwide measures for contact restriction. Now please think about the time after 18 March 2020. Which of the following events have occurred to you? Multiple answers possible’. For the survey of participants in Berlin-Mitte, which had the greatest time gap from the reference date, the following adjustment was made to the above wording: ‘Now please think about the *time of strict measures for contact restriction* after 18 March 2020’.

**Figure 1:**
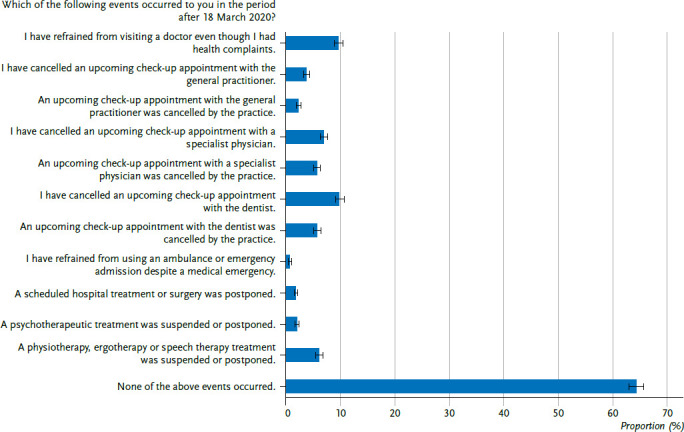
Non-utilisation of health care services (proportions in % with 95% confidence interval, N=7,963) Source: CoMoLo study

The individual events asked about non-utilisation, which could be ticked in each case if they applied, are listed in Figure 1 analogously to their wording and order in the interview. On the one hand, the survey asked about the cancellation of appointments in the various areas of outpatient medical care (GP, specialist, dental care), whereby an explicit distinction was made as to whether appointments were cancelled by the respondent or by the practice. On the other hand, the renunciation of emergency medical care via an ambulance or emergency room as well as the suspension or postponement of a planned hospital treatment, a psychotherapeutic treatment or a therapeutic treatment (ergotherapy, physiotherapy, speech therapy) was queried. In addition, it was asked whether a visit to the physician had been dispensed with despite existing health complaints.

Subsequently, the participants from all four study locations were asked the same question: ‘Have you used telephone or telemedical contact options instead of visiting a medical or psychotherapeutic practice? Multiple answers possible’. The response options were: ‘Yes, by telephone (e.g. telephone consultations)’, ‘Yes, by telemedicine (e.g. video consultations, e-mail contact)’, ‘No’ and ‘No need for examination or treatment’.

#### Disease-specific and sociodemographic factors

The analysis includes information on two of the three central indicators of health status (as components of the internationally established Minimal European Health Module, MEHM [28]): the self-assessment of the general state of health (subjective health) and the presence of chronic diseases or long-term health problems. Subjective health was assessed in the short questionnaire with the following question: ‘How is your health in general?’. The five response options were: ‘Very good’, ‘Good’, ‘Fair’, ‘Bad’ or ‘Very bad’. The presence of chronic diseases or long-term health problems was recorded in the follow-up questionnaire via the following question: ‘Do you have any chronic disease or a long-term health problem? This means diseases or health problems that have lasted or are expected to last for at least 6 months’. The response options included ‘Yes’ and ‘No’.

Furthermore, the participants were asked for detailed information about their medical history based on the following question: ‘Have you ever been diagnosed by a physician with any of the following diseases?’ In a subsequent list, specific questions were asked about individual diseases, with ‘Yes’, ‘No’ or ‘Don’t know’ as possible answers. In this article, information was included from the follow-up questionnaire on ‘heart attack’, ‘circulatory disorders of the heart, narrowing of the coronary arteries, angina pectoris (coronary heart disease)’, ‘heart weakness/heart failure’, ‘atrial fibrillation’, ‘stroke’, ‘high blood pressure/hypertension’, ‘diabetes’, ‘dyslipidemia (elevated fat levels: cholesterol or triglycerides)’, ‘bronchial asthma’, ‘chronic bronchitis, chronic obstructive pulmonary disease (COPD), emphysema (chronic overinflation of the lungs)’, ‘chronic liver disease (liver cirrhosis, liver shrinkage, chronic liver inflammation/hepatitis)’, ‘chronic kidney disease (reduced kidney function, renal insufficiency)’, ‘cancer’, ‘depression’, ‘autoimmune disease (e.g. rheumatoid arthritis, lupus erythematosus, Crohn’s disease, ulcerative colitis, celiac disease, sarcoidosis, multiple sclerosis)’ as well as from the short questionnaire on ‘immunodeficiency (e.g. due to a disease, organ transplant, chemotherapy or taking other drugs such as cortisone)’. The information on the individual physical diseases was used together to define a dichotomous yes/no variable ‘pre-existing physician-diagnosed chronic physical disease’ and the information on depression was used separately to define the dichotomous yes/no variable ‘pre-existing physician-diagnosed depression’.

Using the 2011 version of the International Standard Classification of Education (ISCED [27]), which includes information on school and vocational qualifications from the participants’ short and follow-up questionnaires, the education level was divided into a low (ISCED 1–2), medium (ISCED 3–4) and high (ISCED 6–8) education group.

### 2.3 Study population

The study is based on pooled cross-sectional data from the four study locations (N=9,002). Participants who did not complete a follow-up questionnaire (N=985) and who did not answer the question on non-utilisation of health care services (N=54) were excluded, so that the analysis includes a total of 7,963 participants (N=4,257 women, N=3,706 men). In the further analysis, participants were also excluded who did not answer the question on telemedical contact as an alternative to a visit to a physician’s or psychotherapist’s practice (N=7) or for whom non-plausible information was available (N=83), so that this included a total of 7,873 participants (N=4,205 women, N=3,668 men).

### 2.4 Statistical analyses

In descriptive analyses, the proportions of suspended health care services were determined with 95% confidence intervals (CI). An additional analysis includes stratification by study location and sex.

For a more detailed characterisation of the particular group of respondents who affirmed that they had refrained from visiting a physician despite health complaints, this group was compared with two other groups with regard to sociodemographic and disease-specific factors: 1) with respondents who, in response to the question ‘Have you used telephone or telemedical contact options instead of visiting a medical or psychotherapeutic practice?’, stated that they had no need for an examination or treatment (hereafter referred to as persons without a need for an examination or treatment) and 2) with respondents who did not affirm that they had not visited a physician despite health complaints and did not state that they had no need for an examination or treatment (hereafter referred to as persons who did not refrain from visiting a physician for health complaints). An additional analysis includes logistic regression models with adjustment (control) for place of study, sex, age and education.

All results were calculated taking into account a weighting factor, whereby the respective sample was adjusted to the population structure of the respective municipality with regard to age, sex and educational distribution (at the district level according to the micro-census). In order to take the weighting appropriately into account when calculating confidence intervals and p-values, all analyses were calculated using the survey procedures of SAS 9.4. A statistically significant difference between groups is assumed if the corresponding p-value in the Rao-Scott-Chi-Square test is smaller than 0.05.

## 3. Results

### 3.1 Frequencies of non-utilisation of health care services

Overall, about one-third (35.5%) of respondents reported that they gave up at least one of the surveyed health care services after 18 March 2020 (Figure 1). Among the specialist care services, the most frequent cancellations were of upcoming dental check-up appointments (15.2% in total, 9.8% by the respondents, 5.7% by the practice) and specialist check-up appointments (11.8% in total, 6.9% by the respondents, 5.7% by the practice), followed by the suspension or postponement of physiotherapy, ergotherapy or speech therapy treatment (6.1%) and the cancellation of an upcoming GP checkup appointment (5.8% in total, 3.8% by the respondents, 2.3% by the practice). In comparison, the suspension or postponement of psychotherapeutic treatment (2.0%), the postponement of a planned hospital treatment or operation (1.8%) as well as the renunciation of using an ambulance or emergency room despite a medical emergency (0.7%) were indicated less frequently. The generally formulated question, i.e. not specified according to speciality, about not visiting a physician despite existing health complaints was answered in the affirmative by a total of 9.7%.

This pattern is evident for every study location, even if the respective proportions of non-utilised care services differ slightly between the locations. Moreover, this pattern exists for both sexes, whereby the respective proportions of non-utilisation of almost all health care services are higher for women than for men.

### 3.2 Characteristics of people without a physician visit despite the presence of health complaints

In order to characterise the respondents who refrained from visiting a physician despite health complaints, comparisons were made with respondents without a need for an examination or treatment and with respondents who did not refrain from visiting a physician in the case of complaints.

Respondents who had no need for examination or treatment were, as expected, more likely than the other two groups to rate their health as good or very good, less likely to report having had a chronic disease or long-term health problem for at least six months, less likely to report preexisting physician-diagnosed chronic physical diseases and less likely to report pre-existing physician-diagnosed depression. They were also less often low educated and more often male (Table 2). These differences were also observed after adjusting for place of study, age, sex and education in additionally conducted regression models.

Compared to those who did not refrain from visiting a physician for health complaints, respondents who refrained from visiting a physician for complaints were less likely to rate their health as good or very good, more likely to report a chronic disease or health problem for at least six months and more likely to report pre-existing depression diagnosed by a physician. They were also more often female, under 35 years old, and living in Berlin. Furthermore, respondents who refrained from seeing a physician in the event of health complaints reported more frequently the alternative use of a telephone or telemedical contact option (25.1% by telephone, 6.2% by telemedicine, or 29.1% overall (by telephone or telemedicine)) than those who did not refrain from seeing a physician when they had complaints (14.0% by telephone, 3.2% by telemedicine, or 16.5% overall (by telephone or telemedicine)) (Table 2). In the regression models adjusted for place of study, age, sex and education, the differences between the two groups remained except for place of study.

## 4. Discussion

The present results based on the population-based CoMoLo study show that almost two-thirds of the participating adults from four municipalities in Germany particularly affected by the COVID-19 pandemic did not forego any of the surveyed health care services after the introduction of the containment measures in March 2020. Among the one-third of respondents who forwent at least one of the health care services surveyed, the most frequently reported cancellations of dental and specialist check-up appointments were more than 10% each and almost 10% reported forgoing a visit to the physician despite health complaints. The respondents who refrained from visiting a physician despite health complaints were more often under 35 years of age and female compared to those who did not, and were less likely to rate their health as very good or good, more likely to have a pre-existing diagnosis of depression, and more likely to use a telephone or telemedical contact option.

### 4.1 Classification of the results in comparison to other studies

#### Studies based on population-based survey data

To date, only a few population-based studies have investigated the utilisation of health care services in the context of the COVID-19 pandemic, and each of these studies has focused on different questions and time periods [6, 7].

Results from the online COSMO study, conducted on the basis of repeated and independent cross-sectional samples, indicate that the majority of respondents considered their supply of necessary physician’s visits and medication to be guaranteed during the first pandemic phase. The proportion of people who considered necessary visits at a physician as possible was slightly lower at the beginning of April 2020 in the time frame of strict containment measures (87%) than at the end of July 2020 in the time frame of relaxed measures (94%) [14]. Further results of the COSMO study indicate that the majority of respondents at the end of April 2020 ‘rather not’ to ‘not at all’ agreed to have experienced problems with regard to their medical care in the last four weeks (60%) or to have experienced deteriorations in health due to limitations in medical care (73%) [15]. In contrast, the proportion of respondents who reported in July 2020 that they had postponed cancer screening [29], health check-up [30] or dental appointments [31] due to the COVID-19 pandemic was over 40% each among those with a pending appointment [14], with the majority of postponed dental appointments being checkups [31]. The present study accordingly shows that the majority of respondents reported no cancellations of physician or treatment appointments since the beginning of the pandemic (64%) and that the most frequent cancellations relate to check-up appointments.

A further analysis of the COSMO data shows that the majority of respondents considered their supply of necessary physician’s appointments to be guaranteed also in relation to different care services, whereby the information from the beginning of December 2020 already refers to the second phase of the containment measures. For example, of the 51.7% of respondents with necessary GP or specialist check-up appointments, a total of 3.5% stated that necessary physician’s appointments are currently not possible; of the 9.9% with necessary psychotherapeutic treatment, the figure was 2.0%. It should be noted that the relative proportion of respondents without access to an appointment in relation to those with a required appointment varied for the different care services. The relative proportion was lowest for GP or specialist check-ups and for dental check-ups, each at 6.3%, and highest for psychotherapeutic treatments at 16.5% [16]. Interestingly, in the present study, a pre-existing physician-diagnosis of depression was shown to be an associated factor with the group of people who refrained from visiting a physician despite having health complaints. Overall, this may indicate that people with current or previous mental health problems were most likely to have been affected by not being served by needed health services in the context of the containment measures.

Results from the nationwide GEDA 2019/2020-EHIS study on self-reported utilisation of outpatient GP and specialist services in the last four weeks before the interview indicate a temporary decline during the period of the first phase of containment measures. The proportion of people using a GP in the period from the beginning of April to the end of June 2020 was 30% compared to 38% in the same period of the previous year; the corresponding proportion of people using a specialist was 18% compared to 30% [12]. Another GEDA 2019/2020-EHIS analysis, which included only people with diabetes, also showed a decline in specialist utilisation (24% vs. 43%) but no relevant change in GP utilisation (62% vs. 60%). Possible explanations discussed for this observation were the need for regular GP visits by people with diabetes to care for their condition and adaptations in care provision through telephone consultations [13]. A parallel to the present study can be seen in the fact that the proportion of people who cancelled a specialist check-up appointment (12%) is higher than the proportion of people who cancelled a GP check-up appointment (6%). In addition, the present study also indicates a role for the alternative telemedical contact option among persons who refrained from visiting a physician despite health complaints.

#### Studies based on accounting data

The association of the containment measures with the non-utilisation of health care services can also be seen on the basis of accounting and service data.

An evaluation of the use of outpatient service offered by SHI-accredited physicians and psychotherapists for the months of 2020 in comparison to the corresponding months of the previous year shows a dependence of the observed changes on the measures for contact restriction. Thus, treatment cases declined across all service areas from the beginning of March 2020 (April: -22.7%, May: -15.5%). At the end of May 2020, the number of treatment cases normalised and in June 2020 increases were observed (June: +2.6%), which varied depending on the service area. For most service areas, repeated decreases or approximations of treatment cases to those of the previous year were observed in the third quarter of 2020 (July: -1.3%, August: -0.2%, September: +0.7%) and in the fourth quarter of 2020, after initial increases, declines were again recorded (October: +6.3%, November: -4.5%, December: -3.0%). The outpatient service data show that the declines are particularly pronounced for services that can potentially be postponed, such as screening examinations and training within the framework of the disease management programmes, but are not limited to these. From mid-March onwards, for example, significant decreases in the number of cases were also observed for outpatient emergency and on-call services [8]. A decrease in the number of emergency department visits compared to the previous year – for both outpatient and inpatient stays – is also evident after the introduction of contact restriction measures based on data from 36 university and non-university emergency departments in Germany. Potential reasons discussed were a less frequent occurrence of injuries and accidents as well as infectious diseases, but also a higher inhibition threshold for utilisation (e.g. due to concern about infection with SARS-CoV-2) and a less frequent availability of relatives (who often initiate emergency care, e.g. especially for the elderly) [11]. Here, too, the parallel to the present study can be seen in the fact that decline in utilisation largely, but not exclusively, concerned potentially postponable services. According to the CoMoLo results, 9.7% of the respondents stated that they had not visited a physician despite health complaints and 0.7% that they had not used emergency medical services.

The decrease in treatment cases with direct contact to a physician is offset by a considerable increase in case numbers with telephone counselling (March to December 2020 compared to the corresponding period of the previous year: 6.3 million vs. 3.6 million) and with video consultation (2.5 million vs. around 3,000). This is interpreted as an adjustment of medical and psychotherapeutic services to the necessary care of patients [8]. In line with this, the present study shows that a part of the respondents with examination or treatment needs used telemedical contact options as an alternative and that, as expected, the corresponding proportion among persons who refrained from visiting a physician despite existing health complaints was higher than among persons who did not refrain from visiting a physician in case of complaints (29% vs. 17%).

For inpatient services, according to an evaluation of all hospital stays of AOK-insured persons, there were also decreases, which, compared to the corresponding period of the previous year, were strongest from March to May 2020 (March: -20.9%, April: -35.2%, May: -24.0%), less pronounced in the summer months (June: -6.5%, July: -10.1%, August: -7,8%, September: -6.9%) and stronger again from October to December 2020 (October: -11.4%, November: -16.9%, December: -20.2%) and – similar to the observations based on the population-based data and outpatient accounting data – applied predominantly, but not exclusively, to postponable treatment occasions. In addition to the factors already mentioned in the above paragraph, cited possible influencing factors were regulatory requirements and political recommendations for keeping intensive care capacities available and for postponing hospital stays that can be planned as well as a change in referral behaviour due to reduced consultation times in outpatient practices [9, 10]. In the present study, the postponement of a planned hospital treatment or operation also played a role in a proportion of 1.8% of the respondents.

### 4.2 Strengths and limitations

Overall, the data collected in the CoMoLo study allow a more differentiated view of the utilisation of health care services from a population perspective than was possible in previous population-based studies. With regard to the observations of cancelling in particular – but not exclusively – potentially deferrable services and the relevance of alternative telemedical contact options, results of the present study are in line with results of other studies. In addition, the CoMoLo study integrates further questions, such as on not visiting a physician despite health complaints as well as on sociodemographic and disease-specific factors, which enabled further analyses by linking them.

Nevertheless, when interpreting the results, it should be noted that there were differences between the study sites in terms of infrastructural and sociodemographic characteristics as well as in terms of local outbreak occurrence, which may have resulted in different implemented local containment measures. In addition, the data collection took place at different times in the study locations, so that the time interval to the reference date of 18 March 2020 varies. For the survey in the study location Berlin-Mitte, which had the largest time gap to the reference date, it was therefore additionally specified to think of ‘the time of strict measures for contact restriction’ when answering. The results from additional analyses adjusted or stratified for study location also indicate agreement with the main findings of the analysis with the pooled data from the four study locations. However, the utilisation behaviour in other municipalities, for example in those with less pronounced infection incidence, could have been different, so that the results cannot be directly generalised to the overall adult population of Germany.

Furthermore, the questions integrated in CoMoLo also only allow limited insights into the care needs of the population. For example, in the case of respondents who did not waive health care services, it was not possible to differentiate which proportion used such services or did not plan to use them or did not need them. Further, it was not possible to ask respondents who had cancelled at least one of the requested health care services which specific services they had cancelled (e.g. cancer screening, health check-ups, prenatal care) and why (e.g. fear of contracting SARS-CoV-2, overload in the family). Another limitation for classifying the results is that ‘complaints’ in the question about refraining from visiting a physician despite health complaints, which almost 10% of the respondents affirmed, cannot be characterised in more detail in terms of their type or severity. This is due to the circumstance that the health complaints cannot be clearly assigned to another answer due to the possibility of multiple entries and that no additional free text entries were possible.

### 4.3 Conclusion

In summary, 64% of adults did not forego physician and treatment appointments even under strict contact restrictions in place. However, this was not the case for 36%. Every tenth person even refrained from visiting a physician, although health complaints were present. Compared to persons without such a waiver, these persons were, among other determinants, less often characterised by a good or very good state of health and more often by depression in the past, but more often used telemedical contact options as an alternative to visiting a medical or psychotherapeutic practice.

Longitudinal analyses of possible health effects due to a changed health care situation are still largely lacking for Germany. The experiences of the present and previous studies show how important it is to also analyse changes in care provision and utilisation behaviour caused by external conditions from the perspective of those affected. Only in this way, in combination with results from routine data, conclusions can be drawn as to how care must be designed with regard to crisis situations in order to protect vulnerable groups from care bottlenecks.

## Key statements

- About one third of all respondents refrained from at least one of the health care services surveyed after containment measures began in March 2020.
- Cancellations of dental and specialist check-up appointments were most frequently reported.
- Almost one tenth of the respondents stated that they had refrained from seeing a physician after the start of the containment measures despite health complaints.
- Those who refrained from visiting a physician despite health complaints were more often younger than 35 years and female, reported less often very good or good health and more often a physician-diagnosed depression than those without such a refrain.
- Those who refrained from seeing a physician despite health complaints were more likely to use a telephone or telemedical contact option than those without such a refrain.

## Figures and Tables

**Table 1:** Overview of characteristics of CoMoLo study locations and participants Source: Own table

Kupferzell		Bad Feilnbach	Straubing	Berlin-Mitte
**Chronological order**				
Study period [20–25]	20.05.–09.06.2020	23.06.–04.07.2020	08.09.–26.09.2020	17.11.–05.12.2020
National COVID-19 pandemic phase [4, 26]	Start of summer plateau 2020	Summer plateau 2020	End of summer plateau 2020	Second COVID-19 wave
**Characteristics of the study sites**				
Local spatial structure [25]	Rural	Rural	More urban	Urban
Local infection history before the start of the study [25]	Main infection event: Mid-March to the end of March	Main infection event: Mid-March to mid-April	Main infection event: Beginning of March to the end of May	Diffuse infection event
	Trigger: Local events (mainly church concerts, festivals), travellers returning from ski areas in Austria and Italy	Trigger: Local events (mainly events in the context of local elections, festivals), outbreak in nursing home facility	Trigger: Local events (mainly end of February/beginning of March), outbreaks in nursing home facilities and in the meat processing industry	Trigger: Diverse
**Characteristics of the participants**				
Number of participants [20–25]	2,203	2,152	2,361	2,286
Response rate [20–25]	63%	59%	30%	29%
Age range [24]	18–94 years	18–98 years	18–96 years	18–92 years
Mean age	49 years	51 years	50 years	45 years
Proportion of women [25]	48.5%	50.8%	49.9%	47.4%
Education level (ISCED)				
Low education group	20.6%	18.6%	23.8%	15.7%
Medium education group	49.8%	49.3%	52.6%	34.8%
High education group	29.6%	32.0%	23.6%	49.5%

ISCED = International Standard Classification of Education [27]

**Table 2:** Characteristics of the study population overall and subdivided into groups of people according to their indications of refraining from a physician visit despite health complaints and of a need for examination or treatment (N=7,873) Source: CoMoLo study

Subdivided into groups of persons				
Characteristics	Total (N=7,873)	Persons who refrain from visiting a physician in the event of health complaints (N=708)	Persons who do not refrain from visiting a physician in the event of health complaints (N=3,480)	Persons without need for examination or treatment (N=3,685)
**Sociodemographic factors (proportion in % or mean value, each with 95% CI)**				
Place of study				
Kupferzell	24.6 (23.4–25.9)	24.1 (20.6–28.0)	25.2 (23.5–27.0)	24.1 (22.4–25.9)
Bad Feilnbach	24.2 (23.0–25.4)	21.2 (17.9–25.0)	23.2 (21.6–24.9)	25.7 (24.0–27.4)
Straubing	26.2 (25.0–27.5)	23.9 (20.2–28.0)	27.1 (25.2–29.0)	25.9 (24.1–27.7)
Berlin–Mitte	25.0 (23.7–26.3)	30.8 (26.7–35.2)	24.5 (22.7–26.5)	24.3 (22.5–26.3)
Female sex	49.1 (47.9–50.4)	58.5 (54.1–62.8)	51.5 (49.5–53.5)	45.0 (43.2–46.9)
Age				
18–34 years	27.7 (26.5–29.0)	30.4 (26.4–34.7)	22.8 (21.0–24.6)	32.2 (30.3–34.1)
35–49 years	24.7 (23.5–26.0)	26.0 (22.3–30.1)	24.7 (22.9–26.6)	24.5 (22.9–26.3)
50–64 years	26.5 (25.3–27.6)	24.0 (20.5–27.9)	28.9 (27.1–30.7)	24.5 (22.9–26.2)
65–74 years	10.7 (10.0–11.4)	8.7 (6.8–11.1)	11.9 (10.8–13.1)	9.8 (8.9–10.9)
≥75 years	10.4 (9.6–11.3)	10.9 (8.3–14.2)	11.7 (10.5–13.1)	9.0 (7.9–10.2)
Mean value (in years)	48.7 (48.2–49.2)	47.6 (45.9–49.4)	50.7 (49.9–51.5)	46.9 (46.1–47.6)
Education level (ISCED)^1^				
Low education group	12.8 (11.7–13.9)	16.4 (12.5–21.4)	15.3 (13.7–17.1)	9.5 (8.1–11.2)
Medium education group	50.0 (48.6–51.3)	45.8 (41.5–50.2)	50.6 (48.6–52.6)	50.1 (48.1–52.1)
High education group	37.3 (36.0–38.5)	37.7 (33.9–41.8)	34.1 (32.3–35.9)	40.4 (38.5–42.3)
**Disease-specific factors (proportions in % with 95% CI)**				
Very good/good subjective health^2^	87.8 (86.9–88.7)	73.6 (69.3–77.5)	84.5 (83.0–85.9)	93.7 (92.7–94.6)
Chronic disease/long-term health problem for at least six months^3^	34.3 (33.0–35.6)	47.2 (42.7–51.6)	41.6 (39.7–43.6)	24.6 (22.9–26.4)
Chronic physician-diagnosed physical disease^4^	45.6 (44.2–46.9)	54.0 (49.5–58.5)	51.9 (49.8–54.0)	37.7 (35.7–39.7)
Physician-diagnosed depression^5^	11.2 (10.3–12.1)	24.1 (20.0–28.7)	13.3 (11.9–14.8)	6.8 (5.7–8.0)
**Telephone/telemedical contact (proportions in % with 95% CI)**				
Telephone	8.5 (7.8–9.3)	25.1 (21.6–28.9)	14.0 (12.7–15.5)	-
Telemedical	2.0 (1.6–2.4)	6.2 (3.9–9.5)	3.2 (2.6–4.0)	-

CI = Confidence interval, ISCED = International Standard Classification of Education [27]

In the individual strata, participants with missing values are excluded: 1N = 9, 2N = 185, 3N = 2, 4N = 523, 5N = 205
